# New cell motility model observed in parasitic cnidarian *Sphaerospora molnari* (Myxozoa:Myxosporea) blood stages in fish

**DOI:** 10.1038/srep39093

**Published:** 2016-12-16

**Authors:** A. Hartigan, I. Estensoro, M. Vancová, T. Bílý, S. Patra, E. Eszterbauer, A. S. Holzer

**Affiliations:** 1 Institute of Parasitology, Biology Centre of the Academy of Sciences of the Czech Republic, Branišovská 31, České Budějovice, Czech Republic; 2 Instituto de Acuicultura Torre de la Sal (IATS-CSIC), Castellón, Spain; 3 University of South Bohemia, Faculty of Science, Branišovská 31, České Budějovice, Czech Republic; 4 Institute for Veterinary Medical Research, Centre for Agricultural Research, Budapest, Hungary

## Abstract

Cellular motility is essential for microscopic parasites, it is used to reach the host, migrate through tissues, or evade host immune reactions. Many cells employ an evolutionary conserved motor protein– actin, to crawl or glide along a substrate. We describe the peculiar movement of *Sphaerospora molnari*, a myxozoan parasite with proliferating blood stages in its host, common carp. Myxozoa are highly adapted parasitic cnidarians alternately infecting vertebrates and invertebrates. *S. molnari* blood stages (SMBS) have developed a unique “dancing” behaviour, using the external membrane as a motility effector to rotate and move the cell. SMBS movement is exceptionally fast compared to other myxozoans, non-directional and constant. The movement is based on two cytoplasmic actins that are highly divergent from those of other metazoans. We produced a specific polyclonal actin antibody for the staining and immunolabelling of *S. molnari*’s microfilaments since we found that neither commercial antibodies nor phalloidin recognised the protein or microfilaments. We show the *in situ* localization of this actin in the parasite and discuss the importance of this motility for evasion from the cellular host immune response *in vitro*. This new type of motility holds key insights into the evolution of cellular motility and associated proteins.

Motility is a basic requirement for all cells; one of the main driving forces is acto-myosin contraction which is highly conserved between divergent taxa. Polarized motility is based on the polymerization of actin into filaments leading to compartmentalized pressure that is used to push the leading edge of the cell membrane, and depolymerisation occurs at the trailing edge to create a forward motion^1^. Motility in parasites has been adapted to many functions including, host cell invasion^2^, evasion of the host immune system^3^ and transportation to key tissues within the host^4^. Actin is one of the most conserved proteins in eukaryotes, both the primary sequence and structure have been conserved despite more than 80 actin forms reported in many biological functions in addition to cellular motility^5^. In prokaryotes, the structure and sequence are also somewhat conserved in MreB proteins leading to speculation about a shared origin of actin^6^.

Other common motility proteins such as muscle type myosin heavy chain II have been identified in cnidarians and ctenophores despite lacking striated muscles^7^. It is unclear if this is a case of independent evolution or, reassignment of a protein retained from a bilaterian and cnidarian ancestor with striated muscles. Cnidarians have undergone many unique gene loss and invention events since their evolution from the common ancestor with bilaterians, with a number of taxon or lineage specific gene expansion events within Cnidaria^8^. The innovative nature of their gene evolution could be linked to the highly plastic and variable cnidarian biology in which life strategies range from free living to semi-parasitic and completely parasitic, some with larval stages, some as active migraters etc.^8^. The conquest of these diverse biological niches has led to a high variety of cnidarian orthologues and proteins.

Myxozoa are parasitic cnidarians infecting aquatic vertebrate and invertebrate hosts, which have undergone dramatic morphological simplification and fast radiation since they split from the main lineages of Cnidaria over 500 million years ago^9^. Motility in myxozoan species has been described in a variety of forms and holds key insights into their evolution. *Buddenbrockia plumatellae*, a member of the evolutionary old myxozoan clade Malacosporea, shows tetraradially organized muscle blocks which indicated a shared character between Myxozoa and Cnidarians^10^. The more derived and recent clades of Myxozoa have lost these muscle blocks during morphological reduction and simplification but they have instead evolved other mechanisms and structures for smaller scale single cell movement (spore valve contraction, blebbing, crawling and filopodia etc.)^11^. Amoeboid movement was described as the type of motility responsible for host invasion and within host migration in *Myxobolus cerebralis*^12^, as the infective sporoplasm of the triactinomyxon spore penetrates the fish host epidermis between cells to migrate to the central nervous system^13^. It is assumed that amoeboid movement is common for many myxozoan species as they move from external points of entry (gills, skin, intestine) to their target tissue directly or to the blood stream for transport. Some species show movement in early sporogonic stages within their target tissue such as *Ceratomyxa puntazzi,* which utilizes filopodia to stay afloat in the bile^14^.

*Sphaerospora molnari* is a myxosporean species known from common carp in central Europe^15^. It invades the fish host to produce spores in the epithelia of gills and skin, which are released and presumably ingested by an invertebrate host (presently unknown species). Intriguingly, before *S. molnari* produces spores it circulates as a multicellular blood stage in the fish host. These blood stages are termed “extrasporogonic”, they proliferate in the host for months and are associated with swim bladder inflammation^16^.

Myxozoan blood stages were first reported in carp in 1976 as “unidentified blood objects”^17^ and the movement described as “dancing”; only recently SSU rDNA sequencing identified the cells as *S. molnari*^16^. Their dancing movement has also been described as “twitching”^18^, and is extremely fast, non-directional and produced without tubulin-based cell appendages such as cilia or flagella. This study investigated the molecular motor, its localisation and morphological features enabling this motility. Furthermore, the potential function of such an unusual cellular movement was determined by protein inhibition. This adaptation of conserved proteins into new kinds of motility has evolutionary significance for all metazoan cellular movement.

## Results

The tumbling movement of the blood stages was analysed with real time videos (Supplementary Material 1). Folds on the surface of the primary cell of the parasites give an impulse, causing the cells to rotate around their own axis. The membrane folds are rapidly created and quickly reabsorbed, while new folds emerge in another surface area of the cell. This results in continuous change in the direction of the rotation of the parasite, whilst the cell itself remains “on the spot”. A similar mechanism has not previously been described and we hereby introduce the term Membrane Fold Induced Tumbling (MFIT). In *Sphaerospora molnari* blood stages (herein SMBS), the speed of motility is reduced by increased viscosity of the surrounding medium (1.5% methylcellulose) or by lower temperatures e.g. 5 °C and 10 °C, but neither viscosity increase nor temperature drop hindered fold formation. The shape of the primary cell of SMBS is extremely plastic, it alters with the production of small to large folds in the membrane in addition to the membrane shifting as a whole. The folds periodically emerge from the primary cell and retract quickly (within 0.5–1 second). Supplementary material 1 (video) shows the plasticity and fluid nature of the outer cell. Formation of these folds in the membrane occurs simultaneously on different sides of the cell. The movement is identical in blood stages of different size and variable numbers of secondary/daughter cells within the enclosing primary cell.

**Figure 1:**
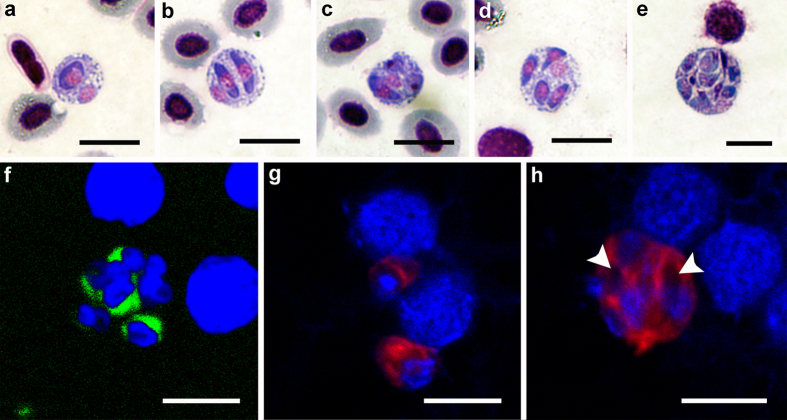
Light and confocal microscopy images of *Sphaerospora molnari.* (**a–e**) Giemsa stained blood stages showing the variety in developmental forms of *S. molnari*. (**f**) Confocal microscopy of parasite stages showing DAPI stained nuclei and α-tubulin (green) within the secondary cells. (**g,h**) Custom *S. molnari* actin 1 antibody (red) showing staining within the primary cell of unicellular parasite stages (**h**) Custom *S. molnari* actin antibody (red) showing staining within the primary cell cytoplasm and not the secondary cells within (arrowheads). Scale bars 10 μm.

SMBS are multicellular and show cell-in-cell development, typical for myxozoans^19^, average measurements are 25.59 (range: 8.44–49.13 μm ; s.d. ± 7.41) in diameter. The number of secondary cells within a primary cell ranges from 1 to 10 (Fig. 1a–e), due to their small size and a minimal cytoplasm, tertiary cells are difficult to distinguish by light microscopy.

Movement is disrupted by the application of Cytochalasin D (actin inhibitor) and 2,3-Butanedione monoxime (BDM; inhibitor of myosin ATPase) which were highly effective at the lowest concentration (0.1 μM) in contrast to the tubulin inhibitor Paclitaxel which had no effect on cell motility even at high concentrations of 10 μM or extended exposure time of 1 hour. Tubulin was visible in *S. molnari* (Fig. 1f) but appeared to be concentrated centrally within the secondary cells rather than peripherally in the primary cell where the folds occur. The actin of *S. molnari* was not visualized using traditional phalloidin staining or general beta actin antibodies however a *S. molnari* specific antibody showed actin labelling concentrated in the outer primary cell cytoplasm (Fig. 1g,h).

**Figure 2:**
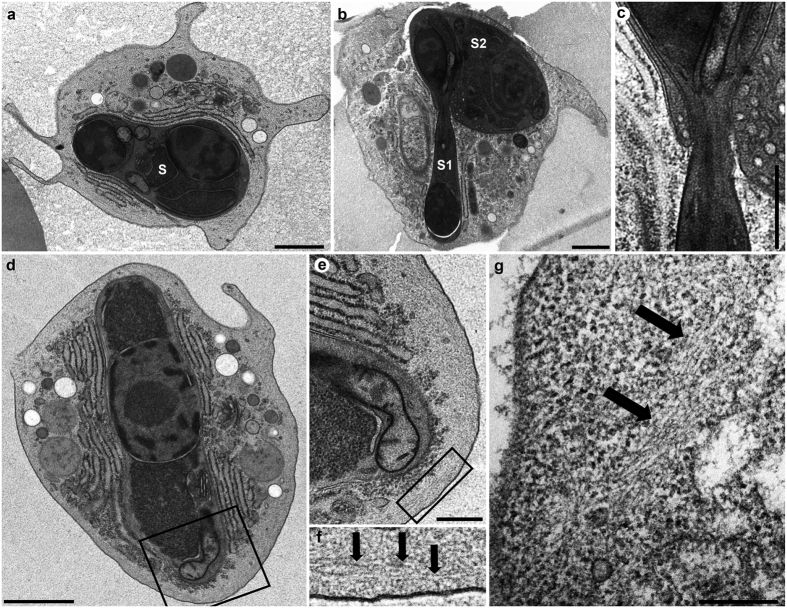
*Sphaerospora molnari* blood stages observed by transmission electron microscopy. (**a**) Primary cell with one secondary (S) and one tertiary cell showing multiple membrane folds all over the cell surface; (**b,c**) Division by cell budding of secondary cell (S1) simultaneous with envolvement by another secondary cell (S2), a single membrane fold on the cell surface; (**c**) Closer view of cell intersection of (**b**); (**d**) Cell filled with ribosomes, actin bundles at the boundary of the primary cell, box indicates view of (**e**); (**e**) Closer view of area with actin bundles, also shows dividing mitochondrion. Box indicates view of (**f**) where actin bundles are present; (**f**–**g**) Actin bundles seen at the periphery of the primary cell indicated by arrows. Scales (**a,b**) = 1 μm; (**c**) = 500 nm; (**d**) = 2 μm, (**e**) = 1 μm, (**g**) = 500 nm.

**Figure 3:**
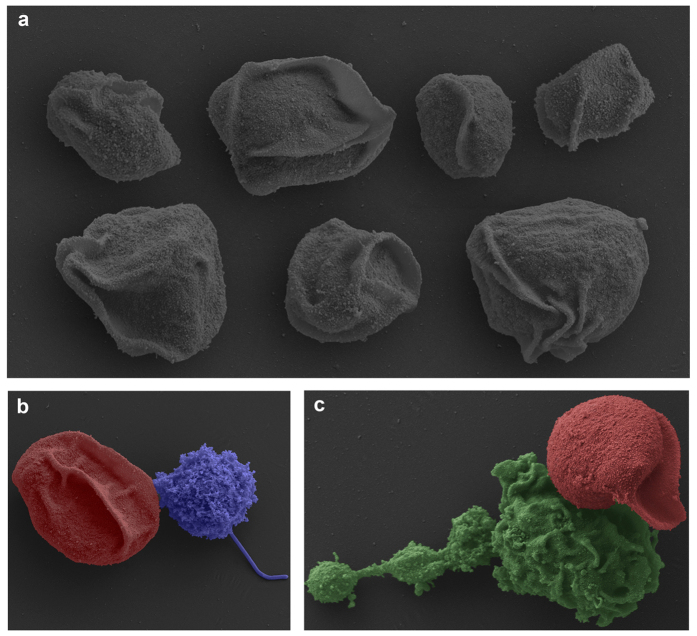
Scanning electron micrographs blood stages of *Sphaerospora molnari*. (**a**) Cells with different surface morphology. **(b**,**c**) Attachment of lymphocyte (blue) and macrophage (green) to blood stage (red). Scale 5 μm.

Transmission electron micrographs from high pressure freeze substitution samples provided a snapshot of the membrane folds in action; they appeared in different conformations all over the surface of the blood stages (Fig. 2a). Key features of myxozoan biology were confirmed including internal double membraned secondary cells, which were commonly filled with electron dense ribosomes and their division (mostly cytokinesis) and reciprocal envolvement occurred simultaneously (Fig. 2b,c). Actin bundles were observed at the edge of the primary cells, not only within the membrane folds themselves (Fig. 2d–f). Bundles were rarely seen within secondary cells, confirmed by tomographic sectioning. Scanning electron microscopy revealed these folds are also visible in the surface of fixed samples (Fig. 3a). SEM also showed contact between SMBS and host immune cells, including lymphocytes (Fig. 3b, lymphocyte blue) and macrophages (Fig. 3c, macrophage green).

**Figure 4:**
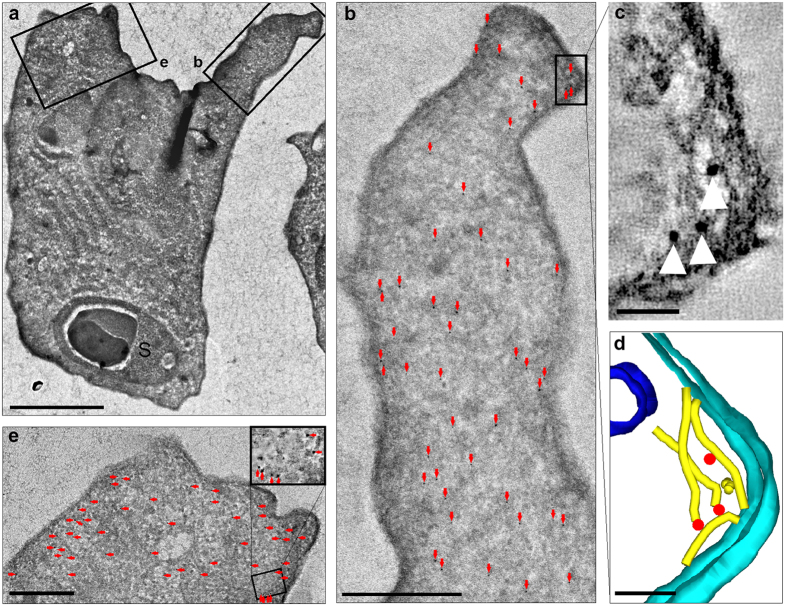
Immunolocalization of *Sphaerospora molnari* actin isoform 1 in cryosections. (**a**) Primary cell with secondary cell (S) at low magnification, two boxes show distribution of labeling within membrane fold extension (**b** and **e**); (**b**) Distribution of immunogold labeling (red arrows) in an extended membrane fold, box indicates view in (**c**) at the extreme tip of the fold; (**c**) Tip of membrane fold showing close view of immunogold labeling on filaments (arrowheads); (**d**) 3D model of (**c**) area with gold nanoparticles (red) in the proximity of suggested actin filaments (yellow), margin of membrane fold i.e. cell membrane (light blue), a vesicle (dark blue). (**e**) Distribution of immunogold labeling at a membrane fold beginning to extend, detail shown in inset.

Immunogold labelling of ultrathin cryosections of *S. molnari* confirmed the presence of actin in the primary cell cytoplasm of *S. molnari* at a higher density (15.3 gold NPs/μm^2^) than the secondary cells (5.2 gold NPs/μm^2^) (Fig. 4a,b,e). Details of statistical evaluation are shown in Supplementary material 2. The presence of specifically labelled actin filaments was confirmed using tomography, these filaments measured 5.56 ± 0.25 nm (n = 7) in diameter (Fig. 4c–e, Supplementary material 3). Despite immunolabeling throughout the primary cell cytoplasm, intact filaments with associated actin specific binding signal were rarely observed in a single section. 3D modeling of tomographic sections was able to show the presence of actin fibres with labelling in the extension of membrane folds (Fig. 4e), also shown in a Z-walk video (Supplementary Material 3).

**Figure 5:**
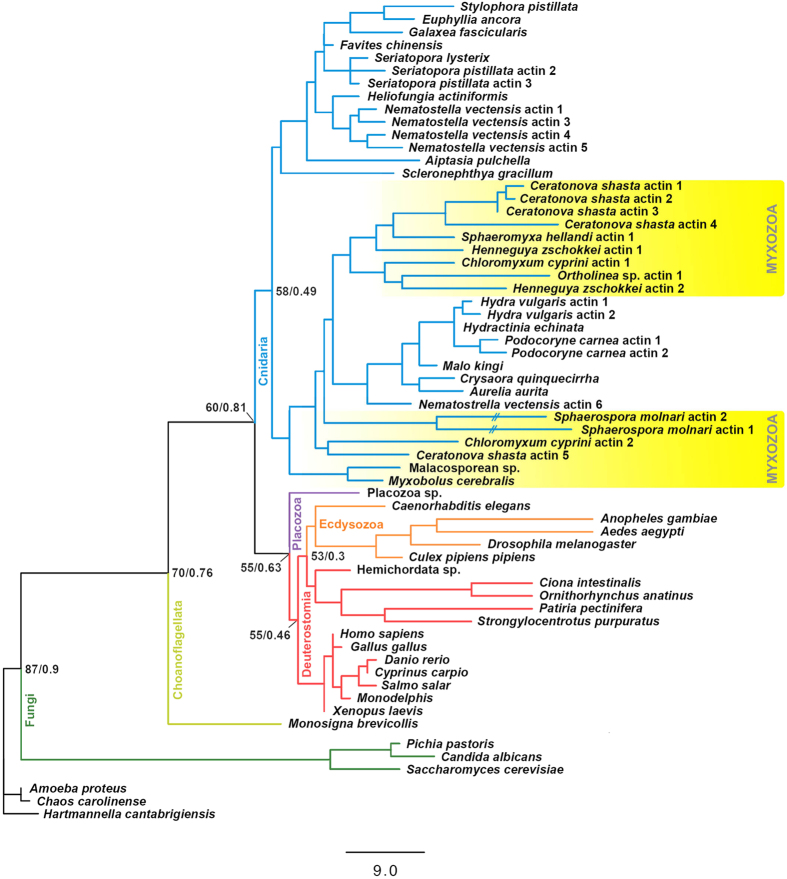
Phylogenetic tree of *Sphaerospora molnari* actins 1 and 2. Maximum Parsimony phylogenetic tree using amino acid sequences of beta actins from representatives of the major metazoan clades, including 23 sequences from free-living cnidarians and 15 from Myxozoa, obtained from genomic data (*C. shasta*, currently unpublished) or PCR amplification (all other sequences). Confidence values at nodes represent percentage of bootstrap replicates (maximum parsimony analysis)/posterior clade possibilities (Bayesian Inference analysis).

Beta actin sequences of six freshwater and marine myxozoan species (*Ceratonova shasta*; *Chloromyxum cyprini*; *Sphaeromyxa hellandi*; *Henneguya zschokkei*; *Ortholinea sp.*) were amplified and sequenced in addition to *S. molnari*. Divergent actin sequences (isoforms) were detected repeatedly within individual species and found to cluster in different phylogenetic subclades (marked in yellow in Fig. 5, *Ceratonova shasta* and *Chloromyxum cyprini*). Two isoforms were found for *S. molnari*, which were highly divergent at the amino acid level, differing between 18–20% from all other taxa’s beta actins, and only sharing 77.9% similarity with each other. Amino acid differences occurred for both actins in all subdomains, the lowest in subdomain 2 and most consistently in subdomain 4 (Supplementary Material 5A–E). The high number of amino acid differences in *S. molnari* actin sequences 1 and 2 was evident from the long branches created in the phylogenetic tree, likely resulting in long branch attraction and their subsequent clustering together, despite large sequence divergence. In the majority of phylogenetic analyses myxozoan actins clustered together with other cnidarian actins, however the inclusion/exclusion of individual non-cnidarian taxa sometimes caused some myxozoan sequences, especially the long branching *S. molnari*, to group with other taxa.

**Figure 6:**
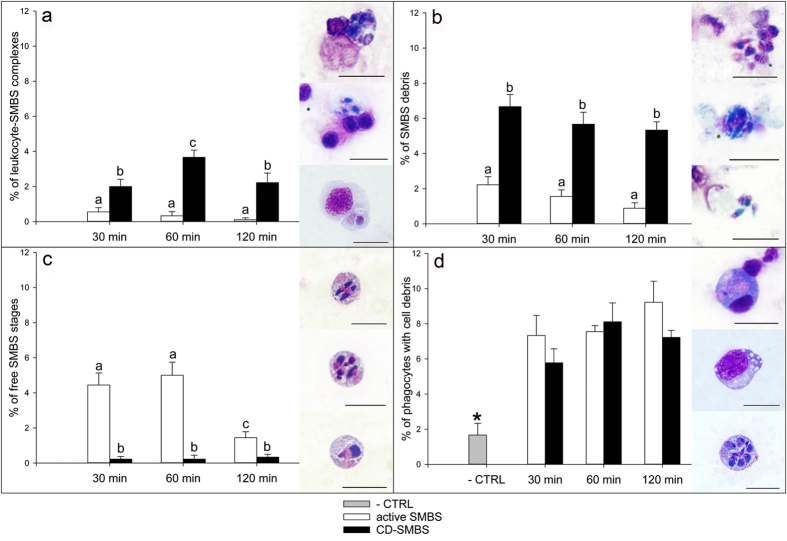
*In vitro* actin motility inhibition assay of *Sphaerospora molnari* blood stages (SMBS) with carp head kidney leukocytes. All graphs show % of cells (SMBS, parasite-SMBS complexes or macrophages) + Standard Error (SE) in a total of 100 cells counted in Giemsa stained smears of carp head kidney leukocytes incubated *in vitro* with SMBS for 30, 60 or 120 min. Motility in CD-SMBS was disrupted by Cytochalasin D treatment previous to the *in vitro* assay. Negative control (−CTRL) consisted of carp head kidney leukocytes alone. (**a**) Cell complexes formed by SMBS and carp leukocytes. (**b**) Lysed SMBS. (**c**) Free, intact SMBS. (**d**) Carp phagocytes with engulfed cell/parasite debris. Representative images of the counted cells are shown for the four graphs. Different letters or asterisks stand for statistically significant differences (*P* < 0.5). Scale bars = 10 μm.

Isolated carp head kidney leukocytes included mainly granulocytes (eosinophils were scarce, neutrophils and basophils were the most abundant ones) and macrophages, as well as lymphocytes. The *in vitro* motility inhibition assay with SMBS showed statistically significant differences between Cytochalasin D inhibited SMBS (CD-SMBS) and active SMBS samples regarding their adhesion to carp leukocytes and their cell integrity (Fig. 6). Cell complexes formed by leukocytes and parasites were significantly more abundant in the CD-SMBS sample than in the sample with active SMBS, at all examined time points (Fig. 6a). Similarly, a higher number of parasite debris, meaning lysed primary cells releasing secondary ones, was found in CD-SMBS compared to active SMBS, at all time points (Fig. 6b). Accordingly, the number of free, viable parasite blood stages was significantly lower in CD-SMBS compared to active SMBS (Fig. 6c). Nevertheless, no differences between the two experimental groups were observed in the numbers of phagocytes with engulfed cell debris (Fig. 6d). In the negative controls, a significantly lower amount of phagocytes contained cell debris and contamination with parasite stages was not observed.

## Discussion

The blood stages of *S. molnari* are an example of new adaptations for old designs; they exhibit a motility mechanism that is unique among all organisms known to date, utilizing the conserved actomyosin machinery with actins that are highly divergent from other described cytoplasmic actins. Cell motility is a highly conserved mechanism across many taxa, however, unseen variations do occur as seen in the unique Membrane Fold Induced Tumbling (MFIT) of *S. molnari*’s blood stages. SMBS movement is rotational but non-directional, cells change position only minorly when in a static medium, and it is unlikely that in a high pressure environment such as the bloodstream this small scale movement is responsible for translocation. Although current and previous^15^ observations on SMBS motility were made *in vitro* (optic microscopy, cell culture) MFIT was also detected in fresh gill and kidney squashes inside small capillaries containing parasite stages (unpublished observations). Some of the fastest cells observed in nature use actomyosin machinery as the basis of their motility, fish keratocytes and stem cells have been recorded at various speeds around 10 μm/min which can be measured by crawling on a substrate from point to point. Unlike amoeboid cells, the movement described here is much faster, substrate-independent and three dimensional at all times.

The MFIT of SMBS constitutes a new model of cell motility based on the following evidence: i) *Lack of leading edge: S. molnari*’s motility is made up of simultaneous expansions and contractions of the outer membrane, therefore there is no “leading” or “trailing edge” as described for cells with amoeboid type motility^1^. ii) *Cell does not move from the spot: S. molnari* does not change its location in wet mount studies^18^ or *in vitro* experiments (present study). This indicates that the parasites are not actively moving in any direction, rather they are subject to the flow dynamics of their environment (i.e. blood flow). iii) *Constant high speed movement*: *S. molnari*’s rapid tumbling movement continues at the same speed indefinitely, the movement can be observed at all times over weeks (up to 26 days) in culture (unpublished data). iv) *Occurs in suspension:* the movement is three-dimensional without any surface contact, in cell culture the blood stages will naturally sink with other host cells but do not attach to the surface of the well or use it for traction.

All sphaerosporid species with blood stages appear to be proliferative but only some are reported to be mobile^20^. Members of other myxozoan genera have been identified in the blood of their vertebrate hosts; most likely the blood is commonly used as a means of transport to the target tissue, e.g. *C. shasta*^21^. In contrast to sphaerosporids, these transport stages are not visible as they are comparatively low in number, very likely small (cell doublets) and immobile. In contrast sphaerosporids proliferate in the blood itself and range in size and number of secondary cells. The effect of Cytochalasin-D and BDM suggests actin and a myosin to be responsible for *S. molnari*’s motility^22^,^23^. There is a high amount of actin within the primary cell cytoplasm as indicated by its intense immunoreactivity to the anti-actin isoform 1 Pab. All of the binding sites reported for phalloidin^24^ were conserved in both *S. molnari* actins yet no reaction was visible whereas cytochalasin-D was able to bind and inhibit actin polymerisation albeit an amino acid change occurred at (reported) binding sites^25^ in both actins. It is possible that the tertiary structures of the actins are responsible for the difference in binding ability between phalloidin and cytochalasin-D, although this requires further investigation. The actin bundles were only seen by immunogold labeling at multiple edges of the primary cell and immunolabeling was higher in the membrane fold extensions providing further evidence that this actin is responsible for the blood stage motility. Similarly, apicomplexan parasites, exhibiting actin-dependent gliding motility, contain actin mostly in an unpolymerized state, which fails phalloidin staining (like SMBS actins), forms short unstable filaments and exhibits a rapid turnover^26^,^27^,^28^. It is tempting to suggest, that SMBS actin isoform 1 exhibits similar kinetic properties enabling the fast MFIT, though involving formation of actin bundles, which are lacking in apicomplexans. In any case, the rapid actin assembly and disassembly and the formation of short unstable filaments could explain our limited ability to visualize SMBS actin filaments. Identification of proteins bound to *S. molnari* actin 1 could provide useful information about the rates of polymerisation within these cells as shown in *Trypanosoma brucei*^29^. The shape and length of the actin filaments could also suggest how the blood stage is able to sustain this rapid movement for such long periods of time on its whole cell surface. Parallel actin bundles have been observed in diverse organisms where they influence the cellular shape (support or stabilization of cellular protrusions or invaginations) and can generate mechanical force for various biological processes (reviewed by ref. 30). The localisation of *S. molnari* actin 1 at the periphery of the cell where the membrane folds occur points to this actin’s involvement in MFIT however, fixed proteins can only tell part of the story; two different kinesin proteins were found localized in *T. brucei*’s flagellum yet knockdown assays showed distinctive functions^31^. Further assays are needed to determine the differential function of *S. molnari’s* two actins.

Actin (crenactin) most likely evolved in the ancestor of bacteria, archaea and eukarya, it has retained sequence and structural features across all groups but its function has diversified^32^,^33^. Various cnidarian actins have been described and are often more related to invertebrate actins and cytoplasmic vertebrate actins rather than any muscle actins^34^,^35^. In a previous study, beta actin isolated from the myxozoan *Myxobolus cerebralis* was placed outside of the Cnidaria, and in fact was placed basal to all Metazoa. Its placement was attributed to the unusually fast evolution of myxozoan protein coding genes^36^. The phylogeny presented here strengthened the position of myxozoans within cnidarians by the addition of several beta actins from this parasite group however, the high sequence divergence of *S. molnari*’s actins makes their position unstable within the tree and resulted in long branches potentially clustered together due to long branch attraction artefacts. The phylogenetic signal of actin is low due to its highly conserved nature^37^, hence the low bootstrap values and some irregularities with regard to the true phylogenetic positioning of some non-cnidarian taxa (position of Ecdysozoa to Deuterostomia in present study). However, while not an exact indicator of *S. molnari*’s phylogenetic position, the phylogenetic tree allows analysis of actin gene evolution and demonstrates the highly probable evolution of myxozoan actins from the forms of their free-living ancestors, in contrast to previous studies^36^. Oddly enough, *S. molnari*’s two actin isoforms show the lowest sequence affinity to each other (77.9% similarity), differing by 77/376-7 amino acids in comparison to the difference between beta actins of *Xenopus laevis* and *Homo sapiens* (2/375 amino acids, see Supplementary material 4). In comparison, human beta actin is 98.7–100% similar to mouse beta actins; 88.8% similar to *Saccharomyces cerevisiae*; and 96.5–97.3% similar to *Nematostella vectensis*. It is possible that unique actin isoforms exist in members of the *Sphaerospora sensu stricto* clade, since this clade is also characterized by large, fully transcribed inserts in the SSU rDNA region^38^. *M. cerebralis* was suggested to have three actin coding genes^36^. Our results indicate the presence of at least two actin isoforms per species (demonstrated for *S. molnari, C. shasta* and *Chloromyxum cyprini*). The similarity even between the isoforms within a species is extreme, for comparison *N. vectensis* actin 1, 3, 4 and 5 are 97.6–98.7% similar to each other in comparison to 95.6–96.3% similarity with the other isoform, actin 6. *C. shasta* actin data were obtained from genome sequences and *C. shasta* actin 1–3 (and potentially 4) likely form the same molecule (expressed genes not known), while the sequence of *C. shasta* actin 5 differs clearly from this group. In contrast, vertebrate actin isoforms are very similar in amino acid sequence, *Fugo rubripes* has only 3 amino acids difference between its cytoplasmic actin isoforms yet all of these are very similar to other vertebrate cytoplasmic actin sequences^39^. The amino acid changes in both *S. molnari* actins are relatively equal in each of the subdomains, with the exception of subdomain 2 (pointed end, approximately amino acids 33–69) that has a third less changes than in the other domains (Supplementary material 4). Skillman *et al*.^40^ demonstrated for the apicomplexan actin of *Toxoplasma gondii* that only a small number of amino acid differences are responsible for filament instability associated to the gliding motility. If the nature of the amino acid substitutions has any impact on the function, post-translational modification or interaction with actin related proteins remains to be seen, however the sheer number of altered amino acids in such a conserved protein should be of interest.

*S. molnari*’s MFIT could be playing a role in the evasion of the host immune system, by allowing the parasite to avoid host immune cell attachment and recognition. Pathogen immune recognition by host cells is a crucial first step to trigger immune mechanisms. During direct cell-to-cell contact, pattern recognition or antigen receptors mediate innate or adaptive immune responses, respectively^41^. Evasion strategies in some fish parasites focus solely on avoiding this recognition event^42^. The tumbling movement of SMBS would minimize contact with immune cells and reduce the chance of recognition and attachment by leukocytes at least *in vitro*. Physical escape either from the extracellular phagosome or the entire phagocyte has been reported for many bacterial and some fungal microorganisms as well as for apicomplexans and haemoflagellates. Such an immune evasion strategy relies on the motility of the microorganism, however, it is usually seen in pathogens with an intracellular life stage inside the host’s phagocytes and occurs after they have been engulfed^43^. Some ciliates show active avoidance behaviour driven by a chemical gradient of antibody concentration in host tissues to elude immobilization^44^, representing a directional response unlike *S. molnari*. The *in vitro* induction of parasite agglutination, phagocytosis or lysis via complement pathways after addition of fish serum has been reported for ciliates^45^, haemoflagellates^46^ and even for myxozoans^47^. In the case of *S. molnari*, the observed higher lysis of CD-SMBS cannot be attributed to a more effective or intensive humoral immune mechanism, since SMBS and CD-SMBS had the same *in vitro* exposure and should have the same susceptibility to humoral factors. Little attention has been drawn to *in vitro* cell-mediated innate mechanisms in fish other than phagocytosis, which remained invariable for both groups in our experiment. However, the incubation of leukocytes with SMBS and CD-SMBS seemed to activate phagocytosis, as the amount of phagocytes with engulfed cell debris was significantly higher in both groups than in the negative control. As parasite stages getting engulfed were rarely observed, their size could be a limiting factor, as previously reported for phagocytes in other fish species^48^,^49^,^50^. Teleost granulocytes are known to bear microbicidal substances like reactive oxygen intermediates, lysosomal enzymes (lysozyme, peroxidases and acid phosphase), biogenic amines (serotonin and histamine), antimicrobial peptides (piscidins), and have been observed degranulating during inflammatory response in host tissues in the presence of diverse pathogens^51^,^52^, including myxozoans^53^. More specifically, degranulation of carp and goldfish neutrophils and their non-specific cytotoxic activity towards bacteria and tumor cells has been reported^54^,^55^,^56^. Innate immune mechanisms are essential in fish when compared with higher vertebrates^57^. Such innate immune mechanisms are likely involved in the observed lysis of SMBS and phagocytosis would be less important than these mechanisms, at least during the first steps of the immune response. The result of this study indicates the importance of the SMBS motility for the avoidance of parasite-leukocyte contact, as significantly higher numbers of CD-SMBS-leukocyte complexes were formed already after 30 min of incubation. Leukocyte contact appears to be essential for subsequent lysis of SMBS, since lysed *S. molnari* primary cells were more abundant in CD-SMBS after incubation with carp leukocytes compared to motile SMBS which were able to avoid recognition and leukocyte attachment. Substrate, parasite behaviour and knockdown assays could provide further insights into the function of this actin in SMBS including non-motility functions. The importance of any motility that avoids the host immune system while the parasite circulates for months within the host blood stream would be critical to its survival.

The new *S. molnari* blood stage cellular motility mechanism represents an intriguing anomaly in the heavily researched field of cell motility, as the distinctive MFIT and the highly divergent cytoplasmic actins. If it represents a long lost ancestral trait or a unique derivative within Myxozoa is a matter for further discussion. However, if this movement aids the blood stages to avoid recognition by immune cells, it would allow “uncontrolled” proliferation in the host and therefore be an important biological trait for myxozoan pathogenicity.

## Methods

All animal procedures were performed under licence number 3831/2013-MZE-17214 in accordance with Czech legislation (Protection of Animals Against Cruelty Act No. 246/1992) and approved by the Czech Ministry of Agriculture. Carp (<2yrs) were obtained from Štrmilov in Czech Republic (49.1644°N, 15.2031°E) and Hortobágy in Hungary (47.3542°N, 21.0000°E) during 2013–2015. *Sphaerospora molnari* blood stages (SMBS) and host white blood cells isolated from whole blood of carp by centrifugation for 5 minutes at 3500 rpm in heparinized hematocrit tubes. Blood smears were made, air dried and fixed/stained with methanol and Giemsa. Measurements were made from 89 blood stages in a single smear using ImageJ^58^.

A custom polyclonal antibody against *S. molnari* actin isoform 1 corresponding to amino acids 315–325 (TKDITGLAAAT) was made by Clonestar Peptide Services to produce a synthetic peptide CTKDITGLAAAT conjugated to BSA. Sera from two rabbits boosted with the peptide in Complete Freud’s Adjuvant four times and affinity column purified was collected.

### Fluorescent confocal microscopy

Cell suspension was left to settle on Superfrost Plus microscope slides (ThermoScientific, Czech Republic), fixed in 4% formalin for 30 min and washed in 1 M PBS. Cells were permeabilised with 1% Triton-X in PBS, and immunolabelled with either α-tubulin monoclonal mouse conjugated with Alexa Fluor 488 [1:200] (Life Technologies, Czech Republic) or primary antibody rabbit anti *Sphaerospora molnari* actin 1 Pab #26 [1:100] (Clonestar Peptide Services, Czech Republic) with secondary goat anti-rabbit IgGAlexa Fluor 594 [1:500] (Life Technologies, Czech Republic). Slides were mounted in Fluroshield with DAPI (Sigma-Aldrich, Czech Republic) and were observed with an Olympus Fluoview 1000 confocal microscope. Negative controls omitting the primary antibodies or the secondary antibody were carried out and were consistently negative.

### Transmission electron microscopy and tomography

50 μl of cells in serum were centrifuged at 1800 RCF, frozen with Leica EM PACT2 high pressure freezer (Leica Microsystems). Using a Leica AFS (Leica Microsystems), samples were freeze-substituted in 100% acetone containing 2% OsO_4_ for 96 hours at −90 °C. Temperature was raised 5 °C/h to −20 °C and after 24 hours samples were rinsed in acetone and infiltrated in graded series of resin (EMBed 812, EMS) solutions (25%, 50% 75% in acetone) 1 hr each. Cells were infiltrated in pure resin overnight, embedded in fresh resin and polymerized at 60 °C for 48 hrs. Ultrathin sections were stained with uranyl acetate and lead citrate and examined either by JEOL 200 kV 2100 F or JEOL JEM-1010 microscopes. Dual-axis tilt series was collected in the range of ±65° with 0.6°-increments using a 200 kV JEOL 2100 F TEM equipped with a high-tilt stage and Gatan camera (Orius SC 1000) and controlled by SerialEM automated acquisition software. Electron tomograms were reconstructed using the IMOD software package. Manual masking of the area of interest was employed to generate 3D surface models.

### Scanning electron microscopy

Cells (50 μl) in serum were fixed in 2.5% glutaraldehyde in 0.1 M phosphate buffer, for 1 hour at 4 °C. Washed cells were left to adhere onto poly-D-lysine (0.1%) coverslips coated for 30 min, then fixed again with 2.5% glutaraldehyde for 15 mins. Stages were post-fixed with 1% OsO_4_, washed in distilled water, dehydrated in a graded acetone series (5 min at each step) and critical-point dried. Coverslips were mounted on stubs, gold sputtered and examined with a FeG-SEM JEOL 7401 F.

### Immunogold labelling

Cell suspensions of host and parasite cells were washed and fixed in 4% formaldehyde/0.1% glutaraldehyde in 0.1 M HEPES for 1 h room temperature; washed in 0.01 M glycine in HEPES; embedded in 10% gelatin at 37 °C, and rotated in 2.3 M sucrose at 4 °C for 4 days. Samples were immersed in liquid nitrogen, ultrathin cryosectioned (Leica EM FCS with Leica UCT cryochamber, Leica Microsystems). Sections were transferred onto Formvar-carbon-coated TEM grids using a drop of 2.3 M sucrose/2% methyl cellulose (1:1). Grids were washed in HEPES, blocked in 5% bovine serum albumin (BSA) and 0.01 M glycine overnight at 4 °C and incubated for 1 h with the custom anti-SMBS actin 1 isoform (1:20) in blocking solution at room temperature. After washing in 0.5% BSA, 0.005 M glycine in HEPES, sections were incubated 1 h in protein A conjugated to 6 nm gold particles (Aurion) diluted 1:40 in the washing solution. Sections were washed in HEPES, distilled water, contrasted and dried using 2% methyl cellulose with 3% aqueous uranyl acetate solution diluted at 9:1. Background labelling was tested by a negative control (in the absence of primary antibody) and were observed by TEM as outlined above.

### Live Video

*Sphaerospora molnari* blood stage motility was recorded with an Olympus Infinity 1–15 C camera on an Olympus BX51 microscope in real time. The cell preparation was a mix of parasite and host *Cyprinus carpio* white blood cells.

Three inhibitors were used, Cytochalasin-D for actin, BDM for myosin ATPases and Paclitaxel for tubulin (Sigma, Czech Republic). Cell suspensions of host and parasite cells were exposed to each of the inhibitors at concentrations of 0.1, 1 and 10 μM in PBS and monitored for 1 hour on microscope slides.

*In vitro* motility inhibition assay was used to analyze the interaction between host immune cells and SMBS. Head kidney homogenate of specific pathogen free carp (n = 2) were obtained by passing the tissue through a 100-μm nylon mesh with RPMI 1640 medium (Sigma, Czech Republic) containing 1% antibiotic/antimycotic solution (10,000 units penicillin, 10 mg streptomycin and 25 ug amphotericin per mL Sigma, Czech Republic), 1% carp serum and 10 U/ml heparin. Cell suspension was layered on 51% (1.072 g/ml) Percoll (GE Healthcare, Czech Republic), centrifuged at 450 RCF for 30 min at 4 °C, leukocytes in the medium/Percoll interface were recovered and washed twice. In order to enhance phagocytic activity, cells were then seeded at 4 × 10^3^ cells/μl in a 96 well plate (50 μl/well in RPMI 1640 complete medium), incubated at 18 °C for 5 days, stimulated with *Escherichia coli* LPS (S0111:B4; Sigma, Czech Republic) (50 μl/well; 100 μg/ml) and incubated for a further 18 h at 18 °C. SMBS (2 × 10^3^ parasites/μl) were obtained (n = 6 carps), half of this sample volume was incubated for 30 min at room temperature with Cytochalasin D (1 μM) to inhibit SMBS motility, washed three times in RPMI 1640 complete medium and then checked for cell viability (0.02 mg/ml propidium iodide/NucBlue; Life Technologies, Czech Republic). Three replicates of the CD-SMBS and three replicates of the active SMBS were each incubated with stimulated head kidney leukocytes (1:1 parasite:leukocyte ratio) in a 96 well plate at room temperature for 2 h (50 μl/well final incubation volume). At 30, 60 and 120 min incubation times, wells were resuspended, three sub-replicates (3 μl) of each well replicate were taken, smeared and Giemsa stained. Smears of stimulated leukocytes were used as negative controls. One hundred cells were counted on each cell smear and numbers of macrophages with engulfed cell material, leukocyte-SMBS complexes, SMBS debris and free SMBS were recorded. Data were analyzed for statistically significant differences between CD-SMBS and active SMBS along time by one-way analysis of variance followed by Student–Newman–Keuls test (significance level *P* < 0.05) (Sigma Stat, SPSS Inc., Chicago, IL, USA).

### Actin Sequencing

Total RNA was isolated from blood stages/host white blood cell mixtures with RNeasy Mini Kit (Qiagen, Czech Republic), for transcriptome sequencing at Beijing Genomics Institute (BGI, Hong Kong) with Illumina HiSeq platform. The Trinity assembly will be published in another publication however the Fragments Per Kb per Million fragments (FPKM) was performed to screen for transcripts with high abundance. Actin transcripts were confirmed in a separate cDNA sample produced by the SMART cDNA Library Construction Kit (Clontech, Czech Republic), by sequencing PCR amplicons produced with degenerate actin primers ACT-F (AAC TGG GAY GAY ATG GAR AAG AT) and ACT-R (ATC CAC ATY TGY TGG AAN GT)^59^. The same primers were used to amplify beta actin sequences from DNA extractions of myxozoan spores belonging to other species (Table 1). PCR conditions were: 5 mins at 94 °C, 30 cycles of 94 °C 1 min, 53 °C for 1 min, and 72 °C for 1 min, with a final extension of 5 mins at 72 °C. Amplicons were cloned into the pDrive Vector (Qiagen, Germany), transformed with TOP10 chemically competent *E. coli* cells (Life Technologies, Czech Republic), and 15 clones per species were sequenced commercially in both directions with M13 primers (https://www.seqme.eu).

### Phylogenetic Analyses

The alignment of beta actins included 66 sequences and 376 AA positions (Supplementary material 5). Maximum Parsimony (MP) analyses were performed in PAUP* v4.b10^60^, using a heuristic search with random taxa addition, the ACCTRAN option, TBR swapping algorithm, all characters treated as unordered, a Ts/Tv ratio of 1:2, and gaps treated as missing data. Bootstraps were based on 1.000 replicates. BI analyses were performed in MrBayes v3.0^61^, using the WAG^62^ model of evolution. Posterior probabilities were estimated from 1.000.000 generations via two independent runs of four simultaneous Markov Chain Monte Carlo algorithms with burn-in set to 10% (100.000 generations).

## Additional Information

**How to cite this article**: Hartigan, A. *et al*. New cell motility model observed in parasitic cnidarian *Sphaerospora molnari* (Myxozoa:Myxosporea) blood stages in fish. *Sci. Rep.* **6**, 39093; doi: 10.1038/srep39093 (2016).

**Publisher's note:** Springer Nature remains neutral with regard to jurisdictional claims in published maps and institutional affiliations.

## Supplementary Material

Supplementary Materials 2 & 5

Supplementary Material 1

Supplementary Material 3

Supplementary Material 4
