# Supplementary material for: New cell motility model observed in parasitic cnidarian Sphaerospora molnari (Myxozoa:Myxosporea) blood stages in fish
Source: Sci Rep. 2016 Dec 16;6:39093. doi: 10.1038/srep39093 (PMC5159882; doi:10.1038/srep39093)
Supplement: Supplementary Materials 2 & 5 [file srep39093-s1.doc]

**Title: New cell motility model observed in parasitic cnidarian *Sphaerospora molnari* (Myxozoa:Myxosporea) blood stages in fish**

Authors: A. Hartigan^1*±^, I. Estensoro^1*^, M. Vancová^1,2^, T. Bílý^1,2^, S. Patra^1, 2^ , E. Eszterbauer^3^, and A.S. Holzer^1^

**Supplemental Data Legends**

**Supplementary material 1**

Live video of *Sphaerospora molnari* blood stages with *Cyprinus carpio* (host) white blood cell preparation. Video was taken in real time with an Olympus Infinity 1- 15C camera on an Olympus BX51 microscope.

**Supplementary Material 2. Immunogold labeling density of Actin isoform 1 within *S. molnari* blood stage’s (SMBS) primary and secondary cells.**

|  | **Primary cell** | | **Secondary cell** | |
| --- | --- | --- | --- | --- |
| **SMBS** | NPs | µm^2^ | NPs | µm^2^ |
| 1 | 40 | 1.41 | 31 | 2.50 |
| 2 | 15 | 2.18 | 10 | 2.90 |
| 3 | 21 | 1.86 | 6 | 3.05 |
| 4 | 18 | 2.21 | 12 | 2.30 |
| 5 | 40 | 1.07 | 15 | 5.83 |
| 6 | 26 | 1.8 | 17 | 2.65 |
| 7 | 56 | 3.6 | 24 | 2.59 |
|  |  |  |  |  |
| Total | 216 | 14.13 | 115 | 21.82 |
| NPs/µm^2^ | 15.28 |  | 5.27 |  |
| StDev | 10.42 |  | 3.52 |  |

NP= nanoparticle

StDev=standard deviation

**Supplementary material 3**

The movie shows a Z walk through the tomographic reconstruction of filaments (yellow) stained by anti – SMBS actin IgG and IgG conjugated to 6 nm gold nanoparticles (green). The dual axis tomogram was reconstructed from a ~37.8 nm thick section divided into 42 slices.

**Supplementary material 4A-E**

Excel spreadsheet of five matrices included for alignment, A- identity (%) and heatmap for complete actin sequences; 2-5- sequence identity (%) and heatmap in each separate subdomains (B-E) of actin.

**Supplementary Material 5**

List of species and accession numbers used in phylogenetic analyses with the original reference.

| **Organism** | **Genbank accession number** | **Reference** |
| --- | --- | --- |
| *Stylophora pistillata* | AGG36339.1 | 1 |
| *Heliofungia actiniformis* | ABD04705 | No publication |
| *Aiptasia_pulchella* | AAQ62633.1 | Unpublished |
| *Seriatopora hystrix* | ADM13664.1 | Unpublished |
| *Stylophora pistillata* | AAR13014 | 2 |
| *Stylophora pistillata* | AGG36337.1 | 1 |
| *Favites chinensis* | BAC44869 | 3 |
| *Scleronephthya gracillimum* | AAT74858 | Unpublished |
| *Nematostella vectensis* | XP_001637076 | 4 |
| *Nematostella vectensis* | XP_001634709 | 4 |
| *Nematostella vectensis* | XP_001630583 | 4 |
| *Nematostella vectensis* | XP_001630069.1 | 4 |
| *Galaxea fascicularis* | BAC44866 | 3 |
| *Euphyllia ancora* | AFP52951 | 5 |
| *Salpingoeca rosetta* | XP_004993570 | Unpublished |
| *Monosiga brevicollis* | AAK27412 | 6 |
| *Placozoa sp. H4* | AGH70113.1 | 7 |
| *Sycon ciliatum* | CCQ18644.1 | 7 |
| *Haliotis iris* | AAX19288.1 | 8 |
| *Caenorhabditis elegans* | CAA34718.1 | 9 |
| *Brugia malayi* | XP_001894819 | 10 |
| *Homo sapiens* | NP_001092 | 11 |
| *Gallus gallus* | CAA25004.1 | 12 |
| *Xenopus (Silurana) tropicalis* | NP_998884.1 | 13 |
| *Danio rerio* | AAH45846.1 | 14 |
| *Cyprinus carpio* | P83750.1 | 15 |
| *Oncorhynchus mykiss* | NP_001117707.1 | 16 |
| *Monodelphis domestica* | XP_001363482.3 | Unpublished |
| *Ciona intestinalis* | XP_002127504.1 | Unpublished |
| *Ornithorhynchus anatinus* | XP_001506759.1 | Unpublished |
| *Myxobolus cerebralis* | AAN86039.2 | 17 |
| *Strongylocentrotus purpuratus* | NP_001119777.1 | 18 |
| *Hydra vulgaris* | XP_002154462.1 | Unpublished |
| *Hydra vulgaris* | XP_002154696.1 | Unpublished |
| *Hydractinia echinata* | ADR10434.1 | 19 |
| *Malo kingi* | ACY74447.1 | 20 |
| *Chrysaora quinquecirrha* | AFS65047.1 | Unpublished |
| *Podocoryne carnea* | P41113.1 | 21 |
|  |  |  |
| *Aurelia aurita* | AGW43036.1 | Unpublished |
| *Anopheles gambiae str. PEST* | XP_321420.4 | 22 |
| *Aedes aegypti* | AAA62350.1 | 23 |
| *Culex pipiens pipiens* | AAY88916.1 | Unpublished |
| *Nematostella vectensis* | XP_001630039 | 4 |
| *Schistosoma mansoni* | P53470.1 | 24 |
| *Amoeba proteus* | AAQ55807.1 | 25 |
| *Chaos carolinense* | AAQ55804.1 | 26 |
| *Hartmannella cantabrigiensis* | AAQ55805.1 | 26 |
| *Candida tropicalis MYA-3404* | XP_002549329.1 | 27 |
| *Saccharomyces cerevisiae S288c* | NP_116614.1 | 28 |
| *Strongylocentrotus purpuratus* | NP_999634.1 | 18 |
| *Drosophila melanogaster* | AAA28321.1 | 29 |
| *Hydra vulgaris* | XP_002154462.1 | Unpublished |
| *Podocoryne carnea* | P41113.1 | 21 |
| *Hydractinia echinata* | ADR10434.1 | 19 |
| *Seriatopora hystrix* | ADM13664.1 | Unpublished |
| *Bombyx mori* | NP_001119726.1 | 30 |
| *Nasonia vitripennis* | XP_001600960.1 | Unpublished |
| *Apis mellifera* | XP_623619.1 | Unpublished |
| *Lepeophtheirus salmonis* | ACO11823.1 | Unpublished |
| *Entamoeba histolytica HM-1:IMSS* | XP_648054.1 | 31 |
| *Dictyostelium discoideum AX4* | XP_636088.1 | 32 |
| *Caenorhabditis elegans*  *ACT-3* | NP_505817.1 | 33 |
| *Acanthamoeba castellanii str. Neff* | XP_004335674.1 | 34 |
| *Saccharomyces cerevisiae S288c* | NP_116614.1 | 35 |

1 Drake, J. *et al.* Proteomic analysis of skeletal organic matrix from the stony coral *Stylophora pistillata* (vol 110, pg 3788, 2013). *Proc Nat Acad Sci U.S.A.* **110**, 7958-7958, doi:10.1073/pnas.1305081110 (2013).

2 Zoccola, D. *et al.* Molecular cloning and localization of a PMCA P-type calcium ATPase from the coral *Stylophora pistillata*. *Biochimica Et Biophysica Acta-Biomembranes* **1663**, 117-126, doi:10.1016/j.bbamem.2004.02.010 (2004).

3 Fukuda, I., Imagawa, S., Iwao, K., Horiguchi, T. & Watanabe, T. Isolation of actin-encoding cDNAs from symbiotic corals. *DNA Res* **9**, 217-223, doi:10.1093/dnares/9.6.217 (2002).

4 Putnam, N. *et al.* Sea anemone genome reveals ancestral eumetazoan gene repertoire and genomic organization. *Science* **317**, 86-94, doi:10.1126/science.1139158 (2007).

5 Shikina, S. *et al.* Germ cell development in the scleractinian coral *Euphyllia ancora* (Cnidaria, Anthozoa). *Plos One* **7**, doi:10.1371/journal.pone.0041569 (2012).

6 King, N. & Carroll, S. A receptor tyrosine kinase from choanoflagellates: Molecular insights into early animal evolution. *Proc Nat Acad Sci U.S.A* **98**, 15032-15037, doi:10.1073/pnas.261477698 (2001).

7 Nosenko, T. *et al.* Deep metazoan phylogeny: When different genes tell different stories. *Mol Phylogenet Evol* **67**, 223-233, doi:10.1016/j.ympev.2013.01.010 (2013).

8 Bryant, M., Flint, H. & Sin, F. Isolation, characterization, and expression analysis of three actin genes in the New Zealand black-footed abalone, *Haliotis iris*. *Marine Biotech* **8**, 110-119, doi:10.1007/s10126-005-5139-5 (2006).

9 Krause, M., Wild, M., Rosenzweig, B & Hirsh, D. Wild-type and mutant actin genes in *Caenorhabditis elegans*. *J Mol Biol* **208**, 381-392, doi:10.1016/0022-2836(89)90503-2 (1989).

10 Ghedin, E. *et al.* Draft genome of the filarial nematode parasite *Brugia malayi*. *Science* **317**, 1756-1760, doi:10.1126/science.1145406 (2007).

11 Sobierajska, K. *et al.* Protein disulfide isomerase directly interacts with beta-actin Cys (374) and regulates cytoskeleton reorganization. *J Biol Chem* **289**, 5758-5773, doi:10.1074/jbc.M113.479477 (2014).

12 Kost, T. Theodorakis, N & Hughes, S. The nucleotide sequence of the chick cytoplasmic beta-actin gene. *Nuc Acid Res* **11**, 8287-8301, doi:10.1093/nar/11.23.8287 (1983).

13 Klein, S. *et al.* Genetic and genomic tools for Xenopus research: The NIH Xenopus initiative. *Dev Dynam* **225**, 384-391, doi:10.1002/dvdy.10174 (2002).

14 Strausberg, R. *et al.* Generation and initial analysis of more than 15,000 full-length human and mouse cDNA sequences. *Proc Nat Acad Sci U.S.A* **99**, 16899-16903, doi:10.1073/pnas.242603899 (2002).

15 Liu, Z. J. *et al.* Isolation and characterization of beta-actin gene of carp (*Cyprinus carpio*). *DNA Sequence* **1**, 125-136 (1990).

16 Ojima, N., Yamashita, M. & Watabe, S. Quantitative mRNA expression profiling of heat-shock protein families in rainbow trout cells. *Biochem Biophy Res Comm* **329**, 51-57, doi:10.1016/j.bbrc.2005.01.097 (2005).

17 Kelley, G. O., Beauchamp, K. A. & Hedrick, R. P. Phylogenetic comparison of the myxosporea based on an actin cDNA isolated from *Myxobolus cerebralis*. *J Eukaryot Microbiol* **51**, 660-663, doi:10.1111/j.1550-7408.2004.tb00605.x (2004).

18 Tu, Q., Cameron, R., Worley, K., Gibbs, R. & Davidson, E. Gene structure in the sea urchin *Strongylocentrotus purpuratus* based on transcriptome analysis. *Genome Res* **22**, 2079-2087, doi:10.1101/gr.139170.112 (2012).

19 Kunzel, T. *et al.* Migration and differentiation potential of stem cells in the cnidarian *Hydra actinia* analysed in eGFP-transgenic animals and chimeras. *Dev Biol* **348**, 120-129, doi:10.1016/j.ydbio.2010.08.017 (2010).

20 Avila-Soria, G. *Molecular aspects of* Carukia barnesi *and* Malo kingi *cDNA expression libraries and screening by antibody probes* PhD thesis, James Cook University, (2009).

21 Aerne, B., Schmid, V. & Schuchert, P. Actin encoding genes of the Hydrozoan *Podocoryne carnea*. *Gene* **131**, 183-192, doi:10.1016/0378-1119(93)90292-B (1993).

22 Mongin, E., Louis, C., Holt, R., Birney, E. & Collins, F. The *Anopheles gambiae* genome: an update. *Trends Parasitol* **20**, 49-52, doi:10.1016/j.pt.2003.11.003 (2004).

23 Ibrahim, M., Eisinger, S. & Scott, A. Muscle actin gene from *Aedes aegypti* (Diptera: Culicidae). *J Med Entomol* **33**, 955-962 (1996).

24 Oliveira, G. & Kemp, W. Cloning of two actin genes from *Schistosoma mansoni*. *Mol Biochem Parasitol* **75**, 119-122, doi:10.1016/0166-6851(95)02508-1 (1995).

25 Fahrni, J. *et al.* Phylogeny of lobose amoebae based on actin and small-subunit ribosomal RNA genes. *Mol Biol Evol* **20**, 1881-1886, doi:10.1093/molbev/msg201 (2003).

26 Nikolaev, S. *et al.* The testate lobose amoebae (order Arcellinida Kent, 1880) finally find their home within Amoebozoa. *Protist* **156**, 191-202, doi:10.1016/j.protis.2005.03.002 (2005).

27 Butler, G. *et al.* Evolution of pathogenicity and sexual reproduction in eight Candida genomes. *Nature* **459**, 657-662, doi:10.1038/nature08064 (2009).

28 Goffeau, A. *et al.* Life with 6000 genes. *Science* **274**, 546-&, doi:10.1126/science.274.5287.546 (1996).

29 Sanchez, F., Tobin, S., Rdest, U., Zulauf, E. & McCarthy, B. 2 Drosophila actin genes in detail – gene structure, protein structure and transcripton during development. *J Mol Biol* **163**, 533-551, doi:10.1016/0022-2836(83)90111-0 (1983).

30 Tavares, J. *et al.* Differential structure of the intronic promoter of the *Bombyx mori* A3 actin gene correlated with silkworm sensitivity/resistance to nucleopolyhedrovirus. *Genet Mol Res* **10**, 471-481, doi:10.4238/vol10-1gmr978 (2011).

31 Loftus, B. *et al.* The genome of the protist parasite *Entamoeba histolytica*. *Nature* **433**, 865-868, doi:10.1038/nature03291 (2005).

32 Eichinger, L. *et al.* The genome of the social amoeba *Dictyostelium discoideum*. *Nature* **435**, 43-57, doi:10.1038/nature03481 (2005).

33 Consortium, C. e. S. & Consortium, C. e. S. Genome sequence of the nematode *C-elegans*: A platform for investigating biology. *Science* **282**, 2012-2018, doi:10.1126/science.282.5396.2012 (1998).

34 Clarke, M. *et al.* Genome of *Acanthamoeba castellanii* highlights extensive lateral gene transfer and early evolution of tyrosine kinase signaling. *Genome Biol* **14**, doi:10.1186/gb-2013-14-2-r11 (2013).

35 Murakami, Y. *et al.* Analysis of the nucleotide sequence of chromosome VI from *Saccharomyces cerevisiae*. *Nature Genetics* **10**, 261-268, doi:10.1038/ng0795-261 (1995).
